# Supplementary material for: Prevalence, Antibiotic-Resistance, and Replicon-Typing of Salmonella Strains among Serovars Mainly Isolated from Food Chain in Marche Region, Italy
Source: Antibiotics (Basel). 2022 May 28;11(6):725. doi: 10.3390/antibiotics11060725 (PMC9219957; doi:10.3390/antibiotics11060725)
Supplement: Supplementary file 1 [file antibiotics-11-00725-s001.zip › antibiotics-1740950-supplementary.pdf]

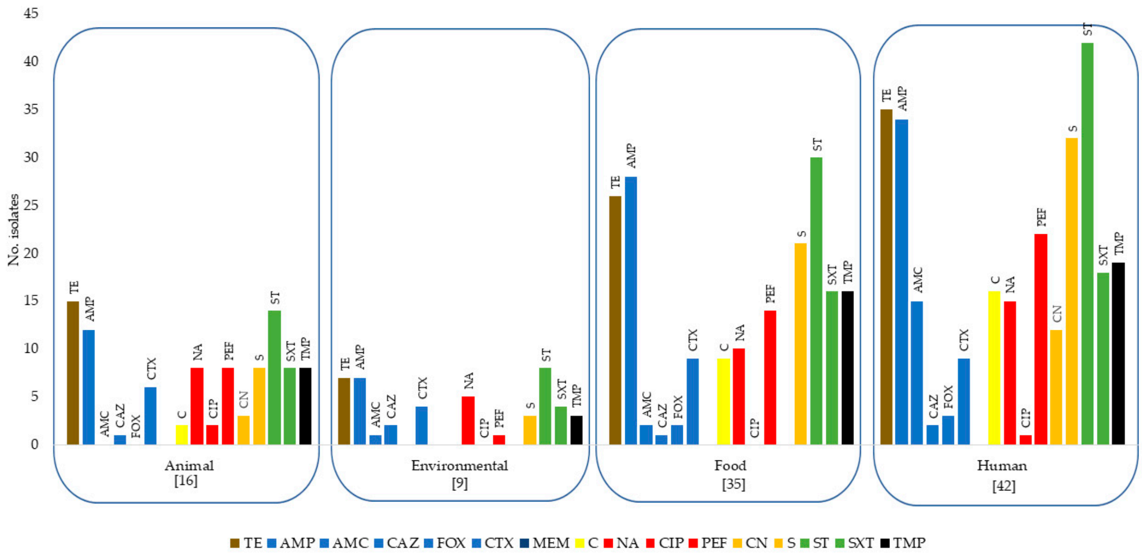

Figure S1. AMR profiles of *Salmonella* strains investigated in this study according to the isolation source. AMP, ampicillin; CTX, cefotaxime; CAZ, ceftazidime; AMC, amoxicillin+clavulanic acid; FOX, cefoxitin; MEM, meropenem; TE, tetracycline; C, chloramphenicol; CIP, ciprofloxacin; NA, nalidixic acid; PEF, pefloxacin; CN, gentamicin; S, streptomycin; SXT, trimethoprim-sulfamethoxazole; ST, sulfisoxazole; TMP, trimethoprim.
